# Perioperative and mid-term outcomes of endovascular aortic repair in patients with moderate-to-advanced chronic kidney disease: a retrospective cohort study

**DOI:** 10.3389/fcvm.2026.1878493

**Published:** 2026-08-07

**Authors:** Yangyang Xia, Chun Ouyang, Xiaoqiang Ye, Mingming Ren, Guang Yang, Lei Huang

**Affiliations:** 1 Department of Cardiovascular Surgery, Peking University Shenzhen Hospital, Peking University, Shenzhen, China; 2 Division of Renal Medicine, Peking University Shenzhen Hospital, Peking University, Shenzhen, China

**Keywords:** aortic pathology, chronic kidney disease, endovascular aortic repair, perioperative complications, retrospective cohort study

## Abstract

**Background:**

Aortic lesions and chronic kidney disease (CKD) frequently coexist, increasing perioperative risks. While endovascular aortic repair (EVAR/TEVAR) offers a less invasive alternative, its safety profile in moderate-to-advanced CKD remains unclear. This study evaluated clinical outcomes and prognostic factors in this high-risk population.

**Methods:**

We conducted a retrospective, age- and sex-matched cohort analysis of 58 patients (29 with CKD, 29 without) undergoing EVAR/TEVAR between 2012 and 2024. Outcomes were compared between groups, with prespecified exploratory subgroup analyses performed within the CKD cohort.

**Results:**

Compared with the non-CKD group, the CKD group had a higher prevalence of hypertension, higher preoperative creatinine and B-type natriuretic peptide levels, lower estimated glomerular filtration rate, and lower hemoglobin levels. Perioperative complications were more frequent in the CKD group (41.38% vs. 6.90%; *p* = 0.006), and postoperative ICU and hospital stays were longer. No 30-day deaths occurred. One-year survival was 96.55% in the CKD group and 100% in the non-CKD group (*p* = 1.000). Only one death was recorded during follow-up, precluding reliable multivariable survival modeling. In the conventional logistic model, CKD was associated with perioperative complications (OR = 9.03, 95% CI 1.63–50.01; *p* = 0.012); in the Firth sensitivity analysis, the estimate was attenuated (OR = 4.40, 95% CI 0.97–19.92; *p* = 0.054).

**Conclusion:**

EVAR/TEVAR may be considered for carefully selected patients with moderate-to-advanced CKD, but these patients experienced a greater perioperative complication burden and longer hospitalization. The survival data were too sparse to support firm comparative conclusions. Individualized risk assessment, perioperative kidney protection, and proactive complication management remain essential.

## 1 Introduction

Aortic lesions, including thoracic and abdominal aortic aneurysms (TAA/AAA), dissection, penetrating ulcer, and intramural hematoma, are life-threatening conditions with a high risk of rupture and mortality (1). Although often asymptomatic, these disorders can deteriorate rapidly, resulting in circulatory collapse or sudden death upon rupture or malperfusion, thus necessitating prompt diagnosis and intervention. Emerging evidence implicates metabolic syndrome, chronic inflammation, and infection in compromising aortic integrity, complicating therapeutic strategies and worsening outcomes (2, 3). While endovascular repair (EVAR; TEVAR) is the preferred minimally invasive treatment (4), its efficacy and long-term prognosis in patients with such comorbidities remain incompletely characterized (5).

Patients with moderate-to-advanced chronic kidney disease (CKD; stages 3–5) frequently present with multiple high-risk comorbidities, including hypertension, atherosclerosis, and metabolic disturbances, predisposing them to aortic pathologies (6). Kidney impairment further elevates the risk of perioperative complications and mortality, necessitating a cautious surgical approach (7). Although kidney dysfunction is a recognized predictor of adverse outcomes in vascular surgery, comprehensive controlled studies evaluating the safety and long-term prognosis of EVAR/TEVAR specifically in this high-risk cohort remain scarce (8–10).

The perioperative course and mid-term outcomes of EVAR/TEVAR in patients with moderate-to-advanced CKD remain poorly defined, and evidence-based management guidelines are lacking (11, 12). To address this gap, we conducted a single-center, retrospective, age- and sex-matched cohort study comparing outcomes between patients with and without moderate-to-advanced CKD. The study aimed to evaluate the procedural safety and clinical outcomes of EVAR/TEVAR in this high-risk population, identify predictors of perioperative complications, and inform individualized treatment strategies.

## 2 Methods

### 2.1 Protocols and ethics

**Figure 1:**
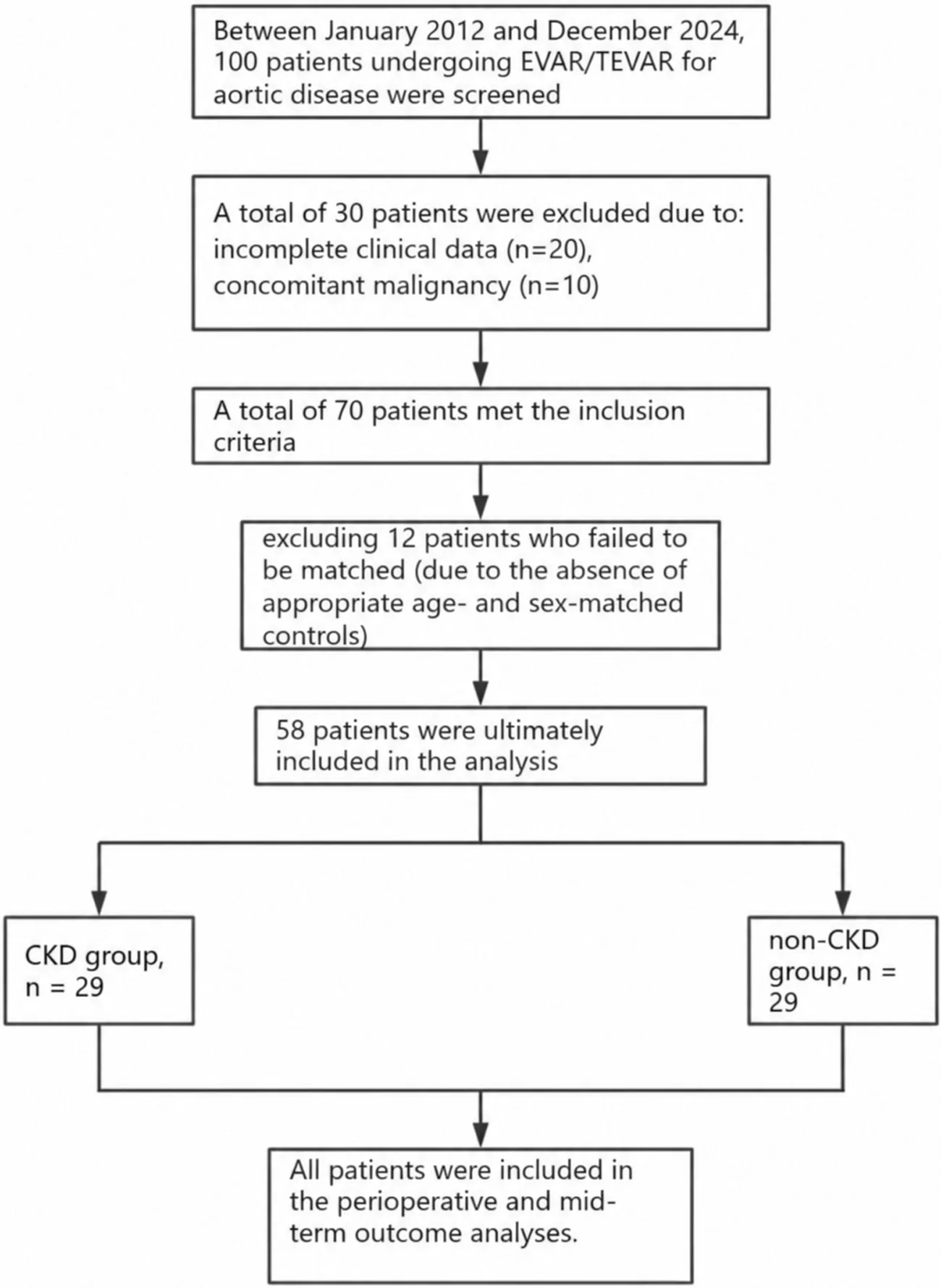
Flowchart of patient enrollment.

This study was a retrospective cohort analysis of 100 patients with aortic lesions who underwent EVAR/TEVAR between January 2012 and December 2024 (Figure 1). Of these, 30 patients were excluded based on predefined criteria: 20 had incomplete clinical data, and 10 had coexisting malignancy. The remaining 70 patients met the inclusion criteria. After excluding 12 patients who could not be matched by age and sex, 58 patients (29 with CKD and 29 without) were included in the final analysis. All enrolled patients were evaluated for perioperative and mid-term outcomes. This study was non-interventional. All procedures adhered to the principles of the Declaration of Helsinki and were approved by the Clinical Research Ethics Committee of Peking University Shenzhen Hospital (2026-107). Written informed consent was obtained from all patients for the use of their data in scientific research.

### 2.2 Inclusion and exclusion criteria

Inclusion criteria: (1) Age ≥18 years; (2) underwent EVAR/TEVAR; (3) CKD group: confirmed diagnosis of CKD stages 3–5 according to KDIGO guidelines (13); (4) availability of complete clinical data and provision of informed consent by the patient or their legal representative for participate in follow-up.

Exclusion criteria: (1) Absolute contraindications to surgery, including severe coagulopathy or anatomical features precluding arterial access for endovascular intervention; (2) presence of advanced malignancy, end-stage liver disease, or other terminal illnesses; (3) incomplete clinical records or refusal to participate in follow-up.

### 2.3 Variables and measurements

#### 2.3.1 Exposure variables

The primary exposure variable was the presence of moderate-to-advanced CKD (CKD group = exposed, non-CKD group = unexposed). Throughout the manuscript, moderate-to-advanced CKD refers to CKD stages 3–5.

#### 2.3.2 Outcome variables

Primary outcomes: (1) incidence of perioperative complications within 30 days after surgery, including pulmonary infection, endoleak, paraplegia, mechanical ventilation lasting more than 48 h, access-site pseudoaneurysm, new postoperative dialysis requirement, acute kidney injury (AKI), and 30-day all-cause mortality; (2) all-cause survival at one year.

For standardized adjudication of adverse events, prespecified diagnostic criteria were used. Pulmonary infection was defined as a new or progressive pulmonary infiltrate on chest imaging in conjunction with compatible clinical features, including fever, purulent sputum, leukocytosis or leukopenia, or worsening oxygenation, together with the requirement for antibiotic treatment during the perioperative hospitalization. AKI was defined according to KDIGO criteria as an increase in serum creatinine of ≥0.3 mg/dL within 48 h, an increase to ≥1.5 times baseline within 7 days, or urine output of <0.5 mL/kg/h for at least 6 h.

Secondary outcomes: (1) duration of postoperative hospital and intensive care unit (ICU) stays; (2) incidence of new complications during follow-up, including recurrent endoleak, new aortic ulceration, pseudoaneurysm rupture, or stent thrombosis; (3) rate of reintervention.

Mid-term outcomes were defined as clinical events and survival outcomes occurring after the perioperative 30-day period and within 5 years after EVAR/TEVAR.

#### 2.3.3 Confounding variables

The following variables were considered potential confounders: demographic characteristics (age, sex, body mass index [BMI]) and comorbidities (hypertension, diabetes mellitus, coronary heart disease [CHD], and hyperlipidemia), preoperative laboratory and echocardiographic markers (serum creatinine, hemoglobin, B-type natriuretic peptide [BNP] and left ventricular ejection fraction [EF]), aortic lesion type (penetrating aortic ulcer, Stanford type B aortic dissection, AAA, and TAA), and procedural factors (emergency vs. elective status, surgical approach, stent type and number, intraoperative blood loss, operative duration, contrast type, and total procedural contrast volume [TPCV]).

#### 2.3.4 Measurement methods

(1) Demographic and comorbidity data were extracted from electronic medical records. (2) Preoperative serum creatinine, hemoglobin, and BNP were measured within one week before surgery using an automated biochemical analyzer (Roche Cobas 8000). BNP was quantified by chemiluminescent immunoassay, and left ventricular ejection fraction was assessed by transthoracic echocardiography (Philips EPIQ 7C). Estimated glomerular filtration rate (eGFR) was calculated using the Chronic Kidney Disease Epidemiology Collaboration equation and combined with the documented clinical history to classify CKD stage according to KDIGO criteria. (3) Aortic computed tomography angiography (CTA) was performed within one week before surgery using a 64-slice dual-source scanner (Siemens Somatom Definition Flash) to characterize lesion location, morphology, and extent. (4) Procedural data included operative urgency, surgical approach, endovascular technique, contrast type and total procedural contrast volume (TPCV), number of stents, blood loss, and operative duration. (5) Follow-up data were collected at 30 days, 3 months, 6 months, and 1 year through outpatient assessments, including CTA, ultrasonography, and laboratory testing, or by structured telephone interviews.

### 2.4 Surgery

#### 2.4.1 Preoperative management

All patients underwent routine preoperative evaluation, including complete blood count, liver and kidney function tests, coagulation profile, and aortic CTA. Elective procedures required an 8 h fasting period. Blood pressure was controlled to below 140/90 mmHg, and fasting blood glucose in diabetic patients was maintained below 8.0 mmol/L. Patients with CKD received perioperative volume management targeting a urine output of 1–2 mL/kg/h. Scheduled dialysis was continued for patients receiving maintenance dialysis.

#### 2.4.2 Surgical procedure

Surgeries were performed by an experienced vascular team (>500 EVAR/TEVAR cases) under general anesthesia via bilateral femoral access. Stent placement was guided by fluoroscopy (Philips AlluraXper FD20), with intraoperative angiography confirming correct positioning, absence of endoleak, and branch vessel patency before closure.

#### 2.4.3 Contrast use and perioperative kidney protection

Because patients with moderate-to-advanced CKD are at increased risk of perioperative kidney injury, kidney protective measures were individualized according to eGFR, urine output, volume status, cardiac function, and dialysis status. In patients without fluid overload, isotonic crystalloid hydration was administered before and/or after EVAR/TEVAR according to the assessed renal risk. Patients with stage 5 CKD or those receiving maintenance dialysis underwent nephrology consultation, and dialysis was performed when clinically indicated to optimize volume and electrolyte balance. Iohexol, a nonionic low-osmolar contrast agent, was used for intraoperative angiography. Contrast exposure was minimized by limiting angiographic acquisitions, using diluted contrast when image quality permitted, and relying on preoperative CTA for procedural planning. Urine output, serum creatinine, and electrolytes were monitored closely after surgery. Loop diuretics were used only to maintain euvolemia or treat fluid overload, not as routine renal prophylaxis. Potentially nephrotoxic agents were avoided whenever feasible, and metformin, renin-angiotensin-aldosterone system inhibitors, and diuretics were adjusted according to kidney function and hemodynamic status. Renal replacement therapy was initiated only when clinically indicated (13–17).

### 2.5 Bias

Selection bias was reduced through age matching (within 3 years), sex matching, and prespecified eligibility criteria. Potential confounding was addressed in multivariable analyses that included clinically relevant comorbidities, anemia, cardiac function, and procedural contrast volume. Outcomes were adjudicated independently by two physicians using prespecified definitions, with discrepancies resolved by consensus. Laboratory and imaging assessments followed standardized institutional protocols. Structured outpatient and telephone follow-up was used to reduce attrition; no patients were lost to follow-up.

### 2.6 Sample size

No *a priori* sample size calculation was performed because the cohort size was fixed by the retrospective design and matching process. Given the small sample and limited number of outcome events, effect estimates are presented with 95% confidence intervals and interpreted as exploratory rather than confirmatory (18).

### 2.7 Statistics

All statistical analyses were performed using SPSS 25.0. Normally distributed continuous variables are presented as mean ± standard deviation (SD) and compared using the independent samples t-test. Non-normally distributed data are shown as median (interquartile range) and analyzed with the Mann–Whitney *U*-test. Categorical variables are reported as number (%) and compared using the *χ*^2^ or Fisher's exact test. Multivariable logistic regression was used to assess the association between CKD and perioperative complications, with results reported as odds ratios (ORs) with 95% confidence intervals (CIs). Hypertension was excluded from the conventional model because all patients with perioperative complications had hypertension, producing quasi-complete separation. A penalized Firth logistic regression model that forced hypertension into the model was used as a sensitivity analysis. Kaplan–Meier curves and the log-rank test were used for descriptive survival comparison. Because only one death occurred, multivariable Cox regression was not performed. Subgroup analyses within the CKD group were stratified by lesion type, CKD stage, and sex and were considered exploratory. All tests were two-sided, and *p* < 0.05 was considered statistically significant.

## 3 Results

### 3.1 Baseline characteristics

Baseline characteristics revealed that the CKD cohort had a significantly higher prevalence of hypertension, elevated preoperative creatinine and BNP levels, and lower hemoglobin concentrations and eGFR compared to the non-CKD group (all *p* < 0.05; Table 1). Conversely, surgical parameters were comparable between groups (all *p* > 0.05; Table 2). Thus, aside from characteristic kidney-related abnormalities, baseline profiles were well matched.

**Table 1:** Comparison of baseline characteristics between the two groups.

Variable	CKD group (*n* = 29)	Non-CKD group (*n* = 29)	Statistic	*p* value
Male, *n* (%)	22 (75.86)	21 (72.41)	*χ*^2^ = 0.000	1.000
Age, years, mean ± SD	65.45 ± 10.88	64.76 ± 10.43	*t* = 0.246	0.806
BMI, kg/m^2^, mean ± SD	23.90 ± 3.96	24.42 ± 3.94	*t* = −0.500	0.619
Smoking history, *n* (%)	11 (37.93)	11 (37.93)	χ^2^ = 0.000	1.000
Comorbidities, *n* (%)				
Hypertension	27 (93.10)	19 (65.52)	χ^2^ = 5.149	0.023*
Diabetes mellitus	8 (27.59)	7 (24.14)	χ^2^ = 0.000	1.000
Coronary heart disease	17 (58.62)	11 (37.93)	χ^2^ = 1.726	0.189
Hyperlipidemia	9 (31.03)	12 (41.38)	χ^2^ = 0.299	0.585
Laboratory parameters				
Preoperative creatinine, μmol/L, median (IQR)	152 (124, 210)	80 (66, 100)	*Z* = 6.018	<0.001*
eGFR, mL/min/1.73 m^2^, median (IQR)	38.27 (28.90, 48.86)	89.40 (69.40, 97.80)	*Z* = −6.438	<0.001*
Hemoglobin, g/L, mean ± SD	112.07 ± 20.10	127.21 ± 15.26	*t* = −3.231	0.002*
BNP, pg/mL, median (IQR)	455 (269, 1,338)	133 (38, 440)	*Z* = 3.717	<0.001*
EF, %, mean ± SD	59.48 ± 8.92	62.76 ± 4.52	*t* = −1.764	0.083

* *p* < 0.05.

BMI, body mass index; BNP, B-type natriuretic peptide; CKD, chronic kidney disease; EF, left ventricular ejection fraction; IQR, interquartile range; eGFR, estimated glomerular filtration rate; SD, standard deviation.

**Table 2:** Comparison of surgical parameters between the two groups.

Variable	CKD group (*n* = 29)	Non-CKD group (*n* = 29)	Statistic	*p* value
Procedure urgency, *n* (%)				
Emergency surgery	11 (37.93)	7 (24.14)	χ^2^ = 0.725	0.395
Elective surgery	18 (62.07)	22 (75.86)		
Surgical approach, *n* (%)				
EVAR	11 (37.93)	15 (51.72)	χ^2^ = 0.627	0.428
TEVAR	18 (62.07)	14 (48.28)		
Endovascular technique, *n* (%)				
Simple covered stent	14 (48.28)	16 (55.17)	χ^2^ = 0.383	0.826
Branched/fenestrated stent	9 (31.03)	7 (24.14)		
Chimney technique	6 (20.69)	6 (20.69)		
TPCV, mL, mean ± SD	104.14 ± 31.57	100.00 ± 28.16	*t* = 0.527	0.600
Intraoperative blood loss, mL, median (IQR)	50 (50, 100)	50 (30, 50)	*Z* = 1.630	0.103
Operative time, min, mean ± SD	107.41 ± 37.31	104.86 ± 44.73	*t* = 0.236	0.814
Number of stents used, median (IQR)	2 (1, 2)	1 (1, 2)	*Z* = 0.542	0.588

CKD, chronic kidney disease; EVAR, endovascular aortic repair; IQR, interquartile range; SD, standard deviation; TEVAR, thoracic endovascular aortic repair; TPCV, total procedural contrast volume.

### 3.2 Perioperative outcomes

Perioperative analysis showed a significantly higher complication rate in the CKD group compared with the non-CKD group (41.38% vs. 6.90%, *p* = 0.006). In the CKD group, observed complications included pulmonary infection (*n* = 7), prolonged mechanical ventilation (>48 h, *n* = 3), endoleak (*n* = 2), paraplegia (*n* = 2), postoperative dialysis (*n* = 3), postoperative AKI (*n* = 1), and puncture-site pseudoaneurysm (*n* = 1). Postoperative hospital and ICU stays were also significantly longer in the CKD group (all *p* *<* 0.05; Table 3). These findings indicate that while EVAR/TEVAR may be a viable option for carefully selected patients with moderate-to-advanced CKD, it is associated with a higher burden of perioperative complications and prolonged hospitalization.

**Table 3:** Comparison of perioperative outcomes between the two groups.

Variable	CKD group (*n* = 29)	Non-CKD group (*n* = 29)	Statistic	*p* value
Perioperative complications, *n* (%)	12 (41.38)	2 (6.90)	χ^2^ = 7.627	0.006*
Pulmonary infection, *n* (%)	7 (24.14)	2 (6.90)	χ^2^ = 2.104	0.147
Endoleak, *n* (%)	2 (6.90)	0 (0.00)	Fisher test	0.491
Paraplegia, *n* (%)	2 (6.90)	0 (0.00)	Fisher test	0.491
Mechanical ventilation > 48 h, *n* (%)	3 (10.34)	0 (0.00)	Fisher test	0.237
Postoperative dialysis, *n* (%)	3 (10.34)	0 (0.00)	Fisher test	0.237
Postoperative AKI, *n* (%)	1 (3.45)	0 (0.00)	Fisher test	1.000
Puncture-site pseudoaneurysm, *n* (%)	1 (3.45)	0 (0.00)	Fisher test	1.000
30-day all-cause mortality, *n* (%)	0 (0.00)	0 (0.00)	—	—
Postoperative ICU stay, days, median (IQR)	1 (1, 3)	1 (1, 2)	*Z* = 1.997	0.046*
Postoperative hospital stay, days, median (IQR)	8 (6, 14)	6 (4, 9)	*Z* = 2.579	0.010*

* *p* < 0.05.

AKI, acute kidney injury; CKD, chronic kidney disease; ICU, intensive care unit; IQR, interquartile range.

### 3.3 Mid-term prognosis

**Figure 2:**
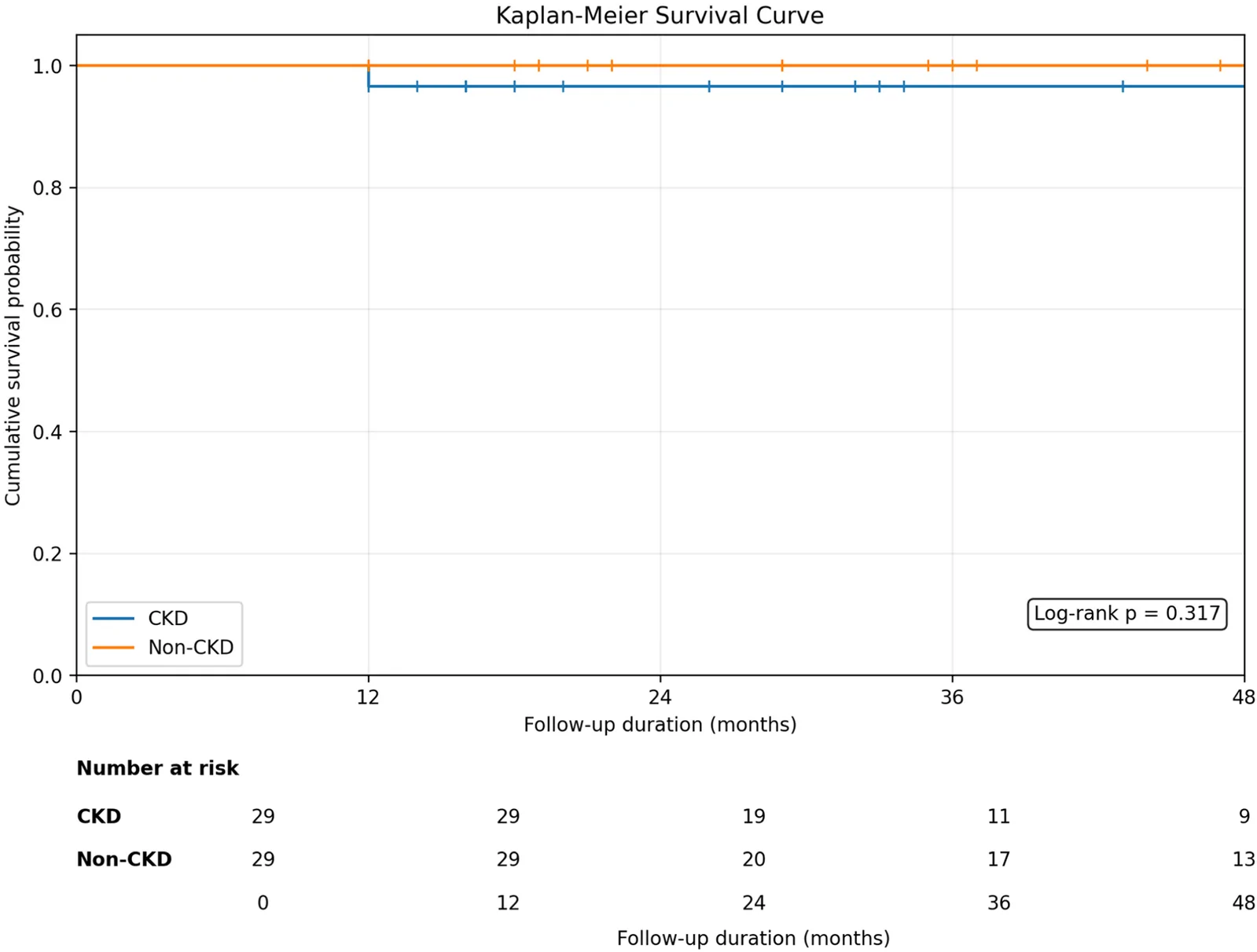
Kaplan–meier survival curve with numbers at risk.

Median follow-up durations were 32 months for the CKD group and 44 months for the non-CKD group (*p* = 0.237). Thirty-day survival was 100% in both groups. No significant differences were observed at one year in survival (96.55% vs. 100%, *p* = 1.000), follow-up complications (17.24% vs. 6.90%, *p* = 0.420), or reintervention rate (6.90% vs. 0%, *p* = 0.491) (Table 4). During follow-up, one death occurred in the CKD group at 12 months, whereas no deaths were recorded in the non-CKD group. Owing to the limited number of events, the Kaplan–Meier analysis did not provide a stable comparative estimate of survival, and the log-rank test showed no significant difference between groups (*p* = 0.317; Figure 2). A multivariable Cox proportional hazards model was not performed because of the insufficient number of outcome events. Overall, mid-term survival after EVAR/TEVAR was comparable between patients with and without moderate-to-advanced CKD in this matched cohort; however, the unequal follow-up durations should be considered when interpreting the survival findings.

**Table 4:** Comparison of mid-term prognosis between the two groups.

Variable	CKD group (*n* = 29)	Non-CKD group (*n* = 29)	Statistic	*p* value
Follow-up duration, months, median (IQR)	32 (18, 52)	44 (22, 61)	*Z* = −1.183	0.237
30-day survival rate, *n* (%)	29 (100.00)	29 (100.00)	—	—
One-year survival rate, *n* (%)	28 (96.55)	29 (100.00)	Fisher test	1.000
Complications during follow-up, *n* (%)	5 (17.24)	2 (6.90)	χ^2^ = 0.650	0.420
Recurrent endoleak, *n* (%)	3 (10.34)	1 (3.45)	Fisher test	0.611
New aortic ulceration, *n* (%)	1 (3.45)	0 (0.00)	Fisher test	1.000
Rupture of pseudoaneurysm, *n* (%)	1 (3.45)	0 (0.00)	Fisher test	1.000
In-stent thrombosis, *n* (%)	0 (0.00)	1 (3.45)	Fisher test	1.000
Reintervention rate, *n* (%)	2 (6.90)	0 (0.00)	Fisher test	0.491

CKD, chronic kidney disease; IQR, interquartile range.

### 3.4 Multivariable and sensitivity analyses

**Figure 3:**
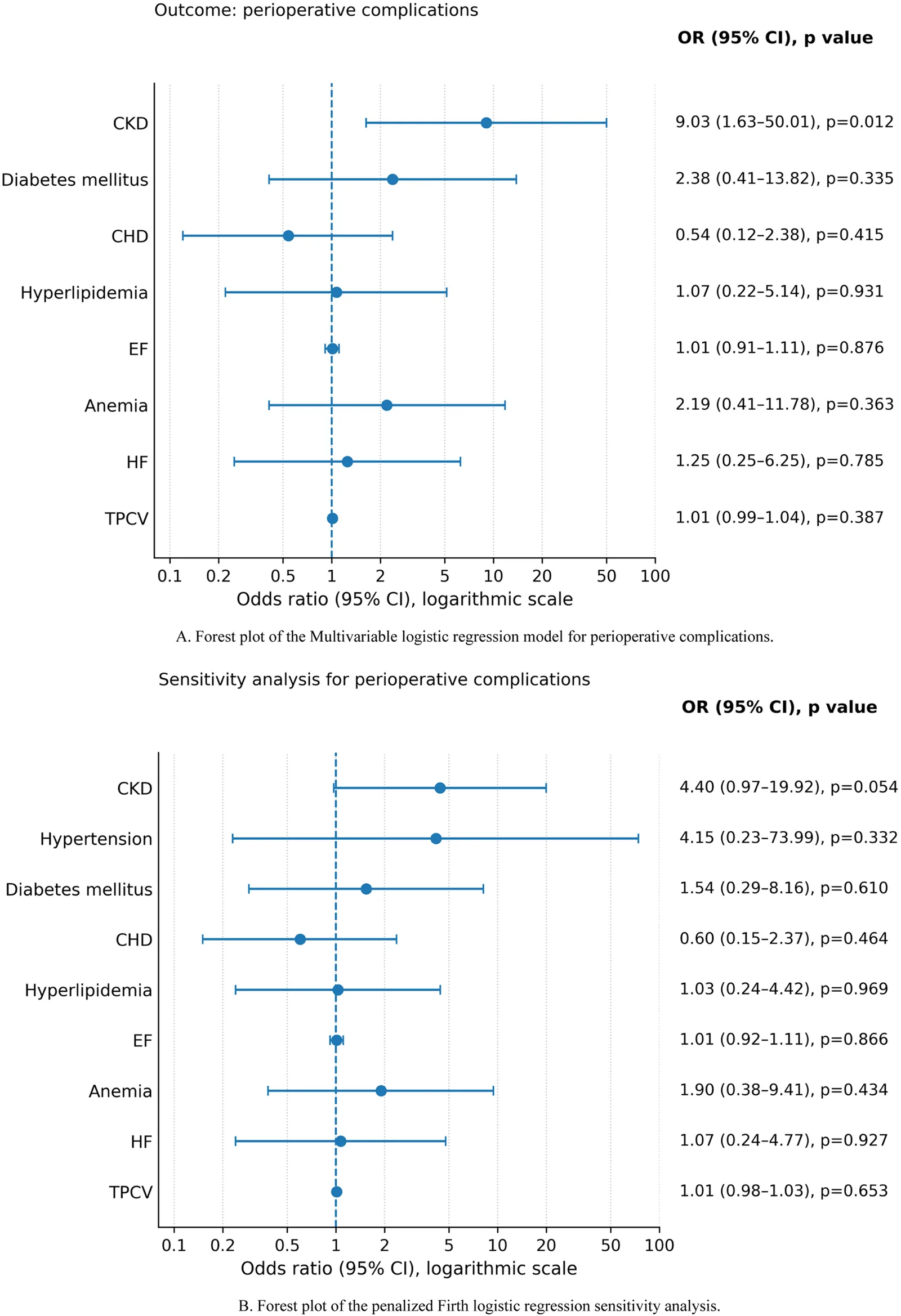
The forest plot of the multivariable logistic regression **(A)** and the sensitivity analysis **(B)**.

Perioperative complications were modeled as the dependent variable, with CKD as the primary exposure. The conventional model adjusted for diabetes mellitus, coronary heart disease, hyperlipidemia, ejection fraction, TPCV, anemia, and heart failure (HF). CKD was associated with perioperative complications in this model (OR = 9.03, 95% CI 1.63–50.01, *p* = 0.012). Hypertension was not included in the conventional model because of quasi-complete separation, as all patients who developed perioperative complications had hypertension (Figure 3A). In the Firth sensitivity analysis, which included hypertension, the CKD estimate remained in the same direction but was smaller and did not meet the conventional threshold for statistical significance (OR = 4.40, 95% CI 0.97–19.92, *p* = 0.054; Figure 3B). Hypertension also showed a positive but imprecise association with perioperative complications (OR = 4.15, 95% CI 0.23–73.99, *p* = 0.332). Together, these findings suggest that CKD was associated with a higher perioperative complication burden. However, the adjusted effect estimates should be considered exploratory because of the limited number of events and model instability related to hypertension.

### 3.5 Subgroup analyses

Subgroup analyses within the CKD cohort revealed no significant differences in perioperative complications, postoperative length of stay, or one-year survival when stratified by lesion type (dissection vs. non-dissection), CKD stage (stage 3 vs. stages 4–5), or sex (all *p* > 0.05; Tables 5–7). Given the small number of patients in several strata, these analyses were underpowered and should be interpreted as exploratory. They should not be taken as evidence that outcomes are equivalent across CKD subgroups.

**Table 5:** Subgroup analysis of the CKD group by lesion type.

Variable	Non-dissection group (Group A, *n* = 21)	Dissection group (Group B, *n* = 8)	Statistic	*p* value
Perioperative complications, *n* (%)	8 (38.10)	4 (50.00)	Fisher test	0.683
Postoperative hospital stay, days, median (IQR)	8 (6, 14)	9.5 (7.5, 12.25)	*Z* = −0.393	0.694
Postoperative ICU stay, days, median (IQR)	1 (1, 2)	2.5 (1, 3.25)	*Z* = −1.305	0.192
One-year survival rate, *n* (%)	20 (95.24)	8 (100.00)	Fisher test	1.000
Complications during follow-up, *n* (%)	4 (19.05)	1 (12.50)	Fisher test	1.000

CKD, chronic kidney disease; ICU, intensive care unit; IQR, interquartile range.

**Table 6:** Subgroup analysis of the CKD group by CKD stage.

Variable	Moderate CKD group (Group C, *n* = 21)	Advanced CKD group (Group D, *n* = 8)	Statistic	*p* value
Perioperative complications, *n* (%)	9 (42.86)	3 (37.50)	Fisher test	1.000
Postoperative hospital stay, days, median (IQR)	8 (7, 11)	8.5 (6, 17.75)	*Z* = −0.270	0.787
Postoperative ICU stay, days, median (IQR)	1 (1, 2)	2 (1, 3)	*Z* = −0.600	0.548
One-year survival rate, *n* (%)	20 (95.24)	8 (100.00)	Fisher test	1.000
Complications during follow-up, *n* (%)	4 (19.05)	1 (12.50)	Fisher test	1.000

CKD, chronic kidney disease; ICU, intensive care unit; IQR, interquartile range.

**Table 7:** Subgroup analysis of the CKD group by sex.

Variable	Male group (Group E, *n* = 22)	Female group (Group F, *n* = 7)	Statistic	*p* value
Perioperative complications, *n* (%)	9 (40.91)	3 (42.86)	Fisher test	1.000
Postoperative hospital stay, days, median (IQR)	8 (5.25, 11)	10 (8.5, 16)	*Z* = −1.387	0.165
Postoperative ICU stay, days, median (IQR)	1 (1, 3)	2 (1.5, 2.5)	*Z* = −0.899	0.368
One-year survival rate, *n* (%)	22 (100.00)	6 (85.71)	Fisher test	0.241
Complications during follow-up, *n* (%)	3 (13.64)	2 (28.57)	Fisher test	0.569

CKD, chronic kidney disease; ICU, intensive care unit; IQR, interquartile range.

## 4 Discussion

The perioperative risks and clinical outcomes of EVAR/TEVAR in patients with moderate-to-advanced CKD remain incompletely defined. In this single-center matched cohort, no perioperative deaths were observed, with 30-day and one-year survival rates of 100% and 96.6%, respectively. Mid-term outcomes were not significantly different from those of non-CKD patients in this cohort. However, the CKD group exhibited significantly higher perioperative complication rates and prolonged postoperative and ICU stays, likely attributable to comorbidities including hypertension, anemia, and reduced organ reserve. Exploratory subgroup analyses did not identify statistically significant differences by lesion type, CKD stage, or sex, but these findings should be interpreted cautiously because of small subgroup sizes. Thus, the present results indicate that EVAR/TEVAR should be individualized for selected patients with moderate-to-advanced CKD, and that generalization to all subgroups is premature.

The greater perioperative complication burden in patients with CKD is biologically plausible. Hypertension, anemia, uremia-associated immune dysfunction, endothelial dysfunction, impaired cardiopulmonary reserve, and a tendency toward volume overload may increase susceptibility to pulmonary infection and delayed respiratory recovery (6, 19–22). Complex vascular anatomy, calcification, landing-zone quality, coagulation abnormalities, and impaired tissue remodeling may contribute to endoleak risk (6, 23). Low baseline renal reserve, perioperative hemodynamic instability, surgical stress, and iodinated contrast exposure may contribute to AKI or a new dialysis requirement. Antoń et al. identified kidney dysfunction, male sex, and aneurysm diameter as predictors of post-contrast AKI after EVAR, supporting integrated assessment of kidney function, anatomy, and contrast burden (14, 23). Spinal cord ischemic injury is multifactorial and may be related to extensive aortic coverage, atherosclerotic burden, and perioperative hypotension (24, 25). These mechanisms were not directly tested in our study and are offered as possible explanations for the observed events. Despite these perioperative risks, comparable mid-term outcomes suggest that EVAR/TEVAR may be feasible in carefully selected patients with moderate-to-advanced CKD when strict case selection, standardized perioperative management, and kidney protective strategies are implemented. Given that only one death was recorded during follow-up, the present study could not support multivariable survival modeling. The limited sample size does not permit firm conclusions, and further exploration of risk factors—particularly overall cardiovascular comorbidity—is warranted in future studies (22, 26, 27). Previous studies have reported increased complications and mortality among patients with CKD stages 4–5 after EVAR/TEVAR (20, 26). In our cohort, mortality rates among patients with CKD stages 4–5 were not significantly higher, but the number of patients in this subgroup was too small to support definitive conclusions (20, 28–30).

The clinical implication is not that EVAR/TEVAR is uniformly safe in CKD, but that it may remain an option when the expected benefit of treating the aortic lesion outweighs renal and systemic risk (26, 29). Preoperative assessment should integrate kidney function, volume status, blood pressure, hemoglobin, cardiac function, and overall comorbidity burden. Perioperative care should emphasize individualized hydration, minimization of contrast exposure, avoidance of nephrotoxins, and early detection of respiratory and kidney complications (20, 31–33). Furthermore, the subgroup analyses do not justify treatment decisions based solely on CKD stage, lesion type, or sex (7).

This study has several limitations. First, the retrospective single-center design and small sample may have introduced selection bias and limited generalizability. Patients with advanced CKD (stages 4–5) and women were underrepresented, and the heterogeneous aortic pathologies differed in acuity, anatomy, and procedural risk. Therefore, analyses specific to individual lesion types and patient subgroups had limited statistical power, and nonsignificant results should not be construed as evidence of equivalence. Second, follow-up was unequal between groups, and the median follow-up of 32 months in the CKD group limited assessment of longer-term survival, kidney-function trajectories, quality of life, and late device-related events. In addition, only one death was recorded during the follow-up period, rendering the survival comparison underpowered and precluding multivariable Cox regression analysis. Third, renal outcomes were incompletely characterized. Only one postoperative AKI event occurred, preventing inferential modeling of AKI, post-contrast AKI, or a dose-response relationship with contrast exposure. Serial postoperative creatinine and eGFR measurements were not uniformly available. Future prospective studies should standardize renal-function time points and documentation of contrast volume, iodine load, kidney protection measures, perioperative hemodynamics, biomarkers, and anatomical variables. Finally, the multivariable estimates were imprecise because of the small number of events and quasi-complete separation for hypertension. Although Firth regression reduced separation-related bias, it could not overcome the limited statistical information. Larger multicenter prospective cohorts are needed to validate these findings and refine risk stratification.

## 5 Conclusions

EVAR/TEVAR may be a feasible treatment option for carefully selected patients with moderate-to-advanced CKD. In this small matched cohort, CKD was associated with more perioperative complications and longer hospitalization, whereas no 30-day deaths occurred and mid-term survival did not differ significantly between groups. These findings should be interpreted as exploratory because of the retrospective single-center design, heterogeneous aortic lesions, unequal follow-up, underpowered subgroup analyses, and imprecise adjusted estimates. Individualized preoperative risk assessment, perioperative kidney protection, and proactive management of respiratory and renal complications are essential.

## Data Availability

The original contributions presented in the study are included in the article/Supplementary Material, further inquiries can be directed to the corresponding author(s).
